# MKRN1 Ubiquitylates p21 to Protect against Intermittent Hypoxia-Induced Myocardial Apoptosis

**DOI:** 10.1155/2021/9360339

**Published:** 2021-08-30

**Authors:** Xue Bai, Hui Yang, Jiayuan Pu, Yan Zhao, Ying Jin, Qin Yu

**Affiliations:** ^1^Department of Pulmonary and Critical Care Medicine, The First Hospital of Lanzhou University, Lanzhou 730000, China; ^2^The First School of Clinical Medicine, Lanzhou University, Lanzhou 730000, China; ^3^Gansu Provincial Maternity and Child Care Hospital, Lanzhou, China

## Abstract

Although chronic intermittent hypoxia- (IH-) induced myocardial apoptosis is an established pathophysiological process resulting in a poor prognosis for patients with obstructive sleep apnea syndrome, its underlying mechanism remains unclear. This study is aimed at exploring the role of makorin ring finger protein 1 (MKRN1) in IH-induced myocardial apoptosis and elucidating its molecular activity. First, the GSE2271 dataset was downloaded from the Gene Expression Omnibus database to identify the differentially expressed genes. Then, an SD rat model of IH, together with rat cardiomyocyte H9C2 and human cardiomyocyte AC16 IH models, was constructed. TUNEL, Western blot, and immunohistochemistry assays were used to detect cell apoptosis. Dihydroethidium staining was conducted to analyze the concentration of reactive oxygen species. In addition, RT-qPCR, Western blot, and immunohistochemistry were performed to measure the expression levels of MKRN1 and p21. The direct interaction between MKRN1 and p21 was determined using coimmunoprecipitation and ubiquitination analysis. MKRN1 expression was found to be downregulated in IH rat myocardial tissues as well as in H9C2 and AC16 cells. Upregulated expression of MKRN1 in H9C2 and AC16 cells alleviated the IH-induced reactive oxygen species production and cell apoptosis. Mechanistically, MKRN1 promoted p21 protein ubiquitination and the proteasome pathway degradation to negatively regulate p21 expression. Thus, MKRN1 regulates p21 ubiquitination to prevent IH-induced myocardial apoptosis.

## 1. Introduction

Obstructive sleep apnea (OSA) syndrome is a disorder mainly characterized by chronic intermittent hypoxia (IH) induced by repeated pharyngeal collapse during sleep. Among middle-aged and elderly people, moderate-to-severe OSA has been reported in 23.4% females and 49.7% males, and it is a well-recognized independent risk factor for cardiovascular disease (CVD) [1]. However, standard OSA treatment, namely, the continuous positive airway pressure system, cannot effectively alleviate major CVDs in OSA patients [2]. It has been shown that increases in IH severity and duration can lead to myocardial apoptosis injury [3], which contributes to CVD complications in OSA patients. Moreover, OSA-related excessive reactive oxygen species (ROS) production, chronic inflammation, endoplasmic reticulum stress, activation of hypoxia-inducible factor 1 (HIF-1) expression, and mitochondrial dysfunction can result in myocardial apoptosis [4]. Among these factors, excessive ROS production induced by a repeated hypoxia-reoxygenation cycle is considered the predominant cause of IH-induced myocardial apoptosis. In addition, ROS serve as signaling molecules to increase cell activation and amplify inflammation, thus forming an amplification loop to further promote ROS production [5]. Consequently, suppressing excess ROS production to reduce IH-induced myocardial apoptosis is considered a promising treatment direction.

Makorin ring finger protein 1 (MKRN1), a highly conserved protein in invertebrates and vertebrates, comprises a C3H zinc finger motif, ring finger motif, and characteristic CyS-His motif, and it is highly expressed in the embryonic nervous system, adult testis, and myocardium [6]. MKRN1 is an E3 ubiquitin ligase [7] that plays important roles in regulating metabolic disorders [8] and tumors [9] through the ubiquitination of substrate proteins. MKRN1 mediates the ubiquitination of AMP-activated protein kinase to promote its proteasome pathway degradation, thus affecting glucose metabolism in the liver and adipose tissues. MKRN1 deletion in mice can significantly suppress diet-induced metabolic syndrome, whereas injection of MKRN1 shRNA into obese mice reverses nonalcoholic fatty liver disease [10]. MKRN1 also mediates the ubiquitination of Fas-associated protein with death domain (FADD). In MKRN1-depleted cervical cancer cells, the FADD content increases, which shows enhanced sensitivity to exogenous apoptosis ligands. In an MDA-MB231 cell xenograft model, MKRN1 deletion results in tumor growth defect after treatment with tumor necrosis factor-related apoptosis inducing ligand (TRAIL), enhancing the sensitivity of triple negative breast cancer to TRAIL treatment [11]. However, the function of MKRN1 in myocardial apoptosis remains unclear.

This study is aimed at investigating the role of MKRN1 in IH-induced myocardial apoptosis and its molecular mechanism to provide a new target for the prevention and treatment of OSA-related cardiovascular complications.

## 2. Materials and Methods

### 2.1. Gene Expression Dataset

The heart gene expression dataset of IH mice (GSE2271) was downloaded from the Gene Expression Omnibus (GEO) database (http://www.ncbi.nlm.nih.gov/geo/) [12]. Then, the transcriptome sequencing data of mice in the control and IH groups were extracted. In addition, the limma R software package was utilized to identify the differentially expressed genes (DEGs) using the threshold values of ∣log_2_ (fold change, FC) | >2 and *p* < 0.05. A heat map was generated using pheatmap R software. We used gene set enrichment analysis (GSEA) software (https://www.broadinstitute.org/gsea/index.jsp) to explore the potential pathways of the DEGs. The statistical differences were determined by the normalized enrichment score and the false discovery rate (FDR) < 0.05.

### 2.2. IH Animal Model

All experiments were conducted in accordance with the guidelines of the Care and Use of Laboratory Animals released by the National Institutes of Health and approved by the Ethics Committee of Lanzhou University First Hospital (no: LDYYLL2020-61). A total of 12 SPF adult male SD rats weighing 230–270 g were provided by the Animal Laboratory Center of Lanzhou University. They were randomly divided into normoxia (NC) and IH groups, with six rats in each group. Both groups of rats were placed into organic glass compartments and provided free access to food and water. For rats in the NC group, compressed air was injected continuously into the compartment, so that the oxygen concentration within the compartment was maintained at 20–21%. The IH model was constructed using the IH control system developed by the Pneumology Department of Lanzhou University First Hospital (patent number: ZL2016200846077) [13]. In brief, nitrogen and compressed air were injected into the compartment for 30 s during the low-oxygen period, so that the minimum oxygen concentration was 5–6%. In the next 10 s, oxygen and compressed air were injected to rapidly increase the oxygen concentration inside the compartment to 20–21%. Later, compressed air was injected for 80 s to maintain the oxygen concentration at 20–21%. The cycle time was 120 s and gas injection lasted 8 h every day for 6 weeks; animals in both groups were placed in ordinary feeding compartments for the remainder of the day. At the end of the exposure period, the left ventricle was isolated from each animal for subsequent detection.

### 2.3. Cell Culture

Human cardiomyocyte AC16 cells (SCC109, Sigma) were cultured in DMEM/F-12 medium (HyClone, Logan, UT) supplemented with 12.5% fetal bovine serum (FBS) (HyClone) and 1% penicillin–streptomycin solution ×100 (Beijing Solarbio Science & Technology Co. Ltd., Beijing, China). Rat cardiomyocyte H9C2 cells and renal 293T cells were purchased from Shanghai Bohu Biotechnology Co. Ltd (Shanghai, China) and cultured in DMEM supplemented with 10% FBS and 1% penicillin–streptomycin solution ×100. All cells were incubated in an incubator (Thermo Fisher Scientific, Waltham, MA, USA) at 37°C with 5% CO_2_.

### 2.4. Cell Transfection

Small interfering RNAs (siRNAs), plasmids, and lentiviral vectors were constructed at ReadyBio Biotechnology Co. Ltd. (Xi'an, China). Using Lipofectamine 2000 (Invitrogen, Carlsbad, CA), the MKRN1-targeting siRNAs (siMKRN1-1: 5′-CGGGATCCTCTCCAACTGCAA-3′, siMKRN1-2: 5′-CAGGCGAAGCTGAGTCAAGAA-3′), or negative control siRNA (siControl) was transfected into cells for 48 h according to the protocols of the manufacturer. The pcDNA3.1-Flag-MKRN1, pcDNA3.1-Myc-p21, and pcDNA3.1-His-Ub plasmids were transfected into 293T cells. Then, Lipofectamine 2000 was used to transfect lentiviral vectors (pLVX-MKRN1-IRES-ZsGreen1, pLVX-p21-IRES-ZsGreen1, and pLVX-IRES-ZsGreen1) and packaging carriers into 293T cells. After 72 h, the supernatant containing the MKRN1-overexpressing and p21-overexpressing lentiviruses was harvested and filtered through a 0.45 *μ*m sterile syringe. Then, H9C2 and AC16 cells were infected with 1 × 10^6^ recombinant lentivirus-transducing units in the presence of 6 *μ*g/ml polybrene (Sigma), with the empty virus vector pLVX-IRES-ZsGreen1 (vector) as the negative control.

### 2.5. *In Vitro* IH Model

When cells reached 70–80% confluency, IH stimulation was conducted as mentioned in a previous study [14]. Cells were subjected to sixteen repeated cycles consisting of hypoxia (1% O_2_ with 5% CO_2_ balanced with N_2_ for 35 min) and normoxia (21% O_2_ with 5% CO_2_ balanced with N_2_ for 25 min).

### 2.6. Histology and Immunohistochemistry (IHC)

For hematoxylin-eosin (HE) staining, the heart tissues were first fixed using 4% paraformaldehyde for 24 h; then, the sections (4 *μ*m) were stained with HE and observed under a microscope. To perform IHC staining, the heart tissue sections (4 *μ*m) were processed with deparaffinage and rehydration according to the manufacturer's instructions. The sections were incubated with primary antibodies at 4°C overnight. The primary antibodies used were MKRN1 (PA5-28461, Invitrogen, California, USA, 1 : 100), p21 (RT1449, H-Bio, Shanghai, China, 1 : 200), Bax (FNab00809, FineTest, Wuhan, China, 1 : 100), Bcl-2 (FNab00839, FineTest, 1 : 100), Bak (FNab00796, FineTest, 1 : 100), caspase 3 (ATA25878, AtaGenix, Wuhan, China, 1 : 200), and glyceraldehyde 3-phosphate dehydrogenase (GAPDH) (FNab03342, FineTest, 1 : 400).

### 2.7. Transferase-Mediated Deoxyuridine Triphosphate Biotin Nick End Labeling (TUNEL) Staining

TUNEL staining was conducted to detect myocardial apoptosis using a kit (Beijing BioRab Co. Ltd., Beijing, China) in line with the manufacturer's instructions. After deparaffinage, the heart tissue sections were treated with protease K (20 *μ*g/ml) for 10 min, followed by 0.3% H_2_O_2_ dissolved in formaldehyde for 10 min and 0.1% Triton X-100 in 0.1% sodium citrate for 2 min. Afterwards, the sections were incubated in the TUNEL reaction mixture. Finally, sections were stained with diaminobenzidine solution at room temperature for 10 min and photos were taken under the microscope. Six fields of view were randomly selected from each tissue section to determine the TUNEL-positive cell percentage, which was calculated by dividing the total number of TUNEL-positive nuclei by the total number of nuclei.

### 2.8. ROS Staining

Dihydroethidium (DHE) staining (Beijing BioRab Co. Ltd., Beijing, China) was conducted to detect the ROS levels in H9C2 and AC16 cells. In the photoprotection environment, cells were incubated with 5 *μ*M DHE at 37°C for 30 min and washed with FBS-free ECM thrice. Under the fluorescence microscope (Leica Microsystems, Wetzlar, Germany), red fluorescence was observed at 535 nm after excitation with blue light and the fluorescence intensity was analyzed using ImageJ software (National Institutes of Health, Bethesda, MD). The cellular ROS level was calculated as the ratio of fluorescence intensity to the baseline level.

### 2.9. Real-Time Quantitative PCR (qPCR)

TRIzol reagent (Invitrogen) was used to isolate and purify total RNA according to the protocols of the manufacturer. Thereafter, cDNA was synthesized using the complementary DNA synthesis kit (Thermo Fisher Scientific). mRNA expression was analyzed with the Maxima SYBR Green/ROX qPCR Master Mix kit (Thermo Fisher Scientific) on an Applied Biosystems (Thermo Fisher Scientific) Prism 7300 detection system, with GAPDH as the endogenous reference. The primer sequences: MKRN1(rat) 5′-ACTGTGGCCGTACTGCCCCTT-3′ (forward), 5′-GCATAGGGGCAAAGCTGCTTC-3′ (reverse); p21(rat) 5′-CGCCGCCGTGATGACCTGGGA-3′ (forward), 5′-CACGTGGTCCTCCGGAGCTGG-3′ (reverse); GAPDH(rat) 5′-GGGAAGCTCACTGGCATGGC-3′ (forward), 5′-GCCGCCTGCTTCACCACCTT-3′ (reverse); MKRN1(human) 5′-GAAGCACCCCTGCAGGGCTCA-3′ (forward), 5′-CTGCAGCATAGGGGCACAGCT-3′ (reverse); p21(human) 5′-GTGATGCGCTAATGGCGGGCT-3′ (forward), 5′-GTCACCCTCCAGTGGTGTCTC-3′ (reverse); GAPDH(human) 5′-ACAACTTTGGTATCGTGGAAGG-3′ (forward), 5′-GCCATCACGCCACAGTTTC-3′ (reverse); the relative gene expression level was calculated using the 2 − ΔΔ*CT* method.

### 2.10. Western Blot (WB) Analysis

The H9C2 and AC16 cells and ventricular tissues were ultrasonically lysed on ice using 100 *μ*l RIPA lysis buffer (containing 50 mM Tris-HCl (pH 7.4), 150 mM NaCl, 1% NP-40, and 2 mM EDTA) supplemented with 1% protease inhibitor. Next, the protein concentration was determined using a BCA Protein Assay Kit (CWBIO, Beijing, China). Then, 30 *μ*g aliquots of total protein samples were separated using SDS-PAGE and transferred onto PVDF membranes. Thereafter, the protein bands were incubated with primary and secondary antibodies. The protein levels were analyzed using the Tanon-5200 Imaging System (Tanon, Shanghai, China). All antibodies used in this assay include MKRN1 (PA5-28461, Invitrogen, 1 : 500), p21 (RT1449, H-Bio, 1 : 1000), Bax (FNab00809, FineTest, 1 : 1000), Bcl-2 (FNab00839, FineTest, 1 : 500), Bak (FNab00796, FineTest, 1 : 1000), caspase 3 (ATA25878,AtaGenix, 1 : 500), GAPDH (FNab03342, FineTest, 1 : 500), His (K10846, BioRab, 1 : 200), and anti-Lys 48 UB (P4D1, Santa Cruz, CA, 1 : 200). Moreover, the ECL kit (Pierce Chemical Co., Rockford, IL) was used for protein visualization and the protein gray level was detected using ImageJ software version 1.46.

### 2.11. Co-Immunoprecipitation (co-IP) Assay

Cells were washed with cold phosphate-buffered saline solution thrice and lysed in a buffer solution (consisting of 50 mM Tris-HCl (pH 7.5), 150 mM NaCl, 0.5% Triton X-100, and 1 mM EDTA) supplemented with a protease inhibitor cocktail. Then, the lysate was incubated with p21 antibody (RT1449, H-Bio, 1 : 1000), Myc antibody (FNab05462, FineTest, 1 : 1000), or normal IgG antibody for 2 h or overnight at 4°C, and then incubated with protein G Sepharose (GE Healthcare, Buckinghamshire, UK) at 4°C for 2 h to conduct the CO-IP assay. The immunoprecipitate was boiled for 5 min in ×2 sample buffer.

### 2.12. *In Vivo* Ubiquitination Analysis

The ubiquitination assay was conducted under denaturing conditions to detect the ubiquitinated endogenous and upregulated p21 protein expression. In brief, to detect the His-Ub-ubiquitinated protein or the endogenously ubiquitinated protein under the denaturation condition, proteins were boiled in phosphate-buffered saline containing 1% SDS and 5 mM N-ethylmaleimide for 10 min to lyse the cells. Then, the lysate was immunoprecipitated using Myc antibody or p21 antibody in the lysis buffer (final concentration, 0.1% SDS). Next, the proteins were transferred onto PVDF membranes and denatured with 6 M guanidine-HCl (20 mM Tris-HCl (pH 7.5), 5 mM mercaptoethanol, and 1 mM phenylmethyl sulphonyl fluoride) for 30 min at 4°C. The ubiquitinated proteins were detected using UB antibody or His antibody.

### 2.13. Statistics and Data Analysis

All statistical analyses were performed using SPSS 22.0 software. Quantitative variables are expressed as mean ± standard deviation. First, the normality test was performed. For data conforming to normal distribution with homogeneity of variance between groups, the Student's *t*-test was adopted to detect the significant differences between two groups, whereas one-way ANOVA and Bonferroni post hoc tests were applied when comparing the significant differences among multiple groups and between two groups. Data not satisfying the abovementioned conditions were analyzed using the nonparametric Mann–Whitney *U* test. Statistical significance was set at *P* < 0.05.

## 3. Results

### 3.1. Bioinformatics Prediction

**Figure 1:**
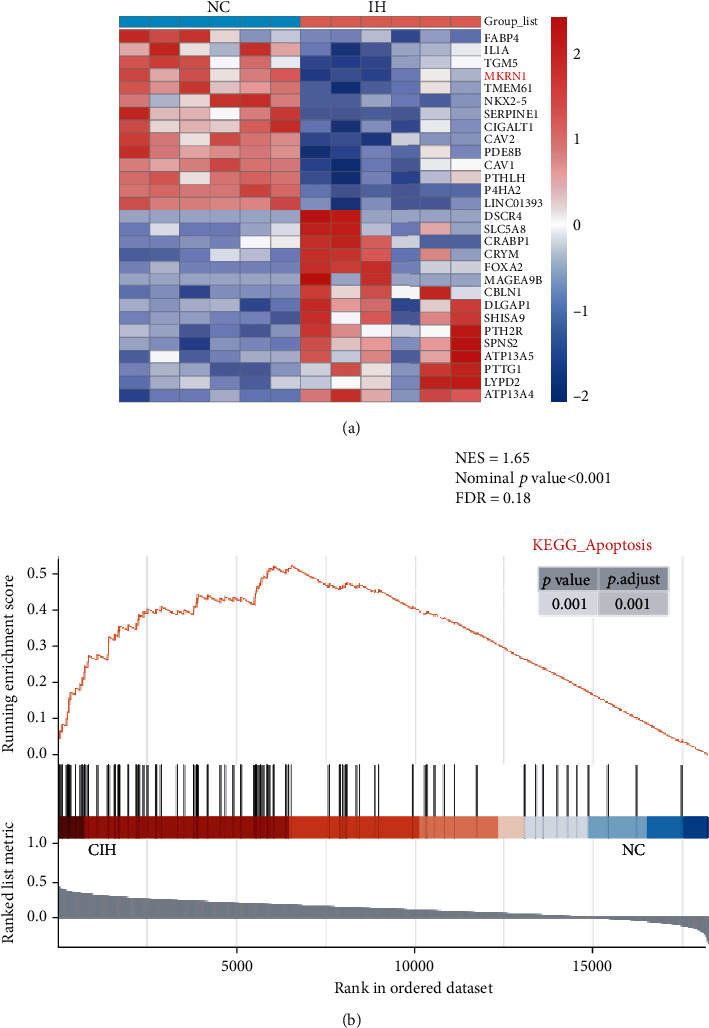
Bioinformatics prediction results. (a) Heat map showing the expression of differentially expressed genes in mouse myocardial tissues between the NC and IH groups; MKRN1 expression was downregulated in IH myocardial tissues; (b) GSEA showed that MKRN1 was closely related to apoptosis.

To discover the DEGs in IH myocardial tissues, we analyzed the GSE2271 dataset [12]. Compared with myocardia of the NC group, MKRN1 mRNA expression significantly decreased in the myocardial tissues of IH mice (Figure 1(a)).

The samples were divided into low and high MKRN1 expression groups. After GSEA, the abnormal expression of MKRN1 was closely related to cell apoptosis (Figure 1(b)). Therefore, we speculated that MKRN1 was related to IH-induced myocardial apoptosis.

### 3.2. IH Induced Myocardial Apoptosis and Reduced MKRN1 Expression

**Figure 2:**
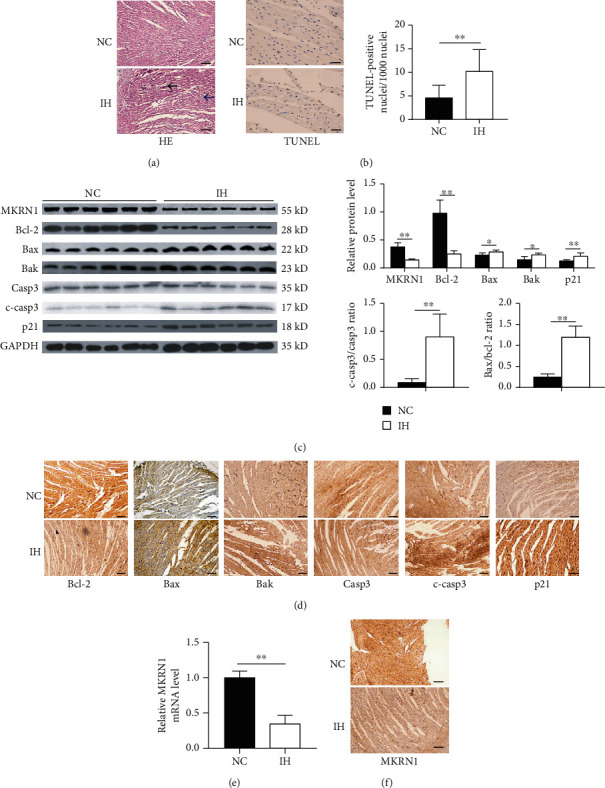
IH promotes myocardial apoptosis and downregulates MKRN1. (a) HE staining of heart tissue sections (scale bar, 100 *μ*m); the blue arrow shows myocardial structure being disorganized; the black arrow shows infiltration of inflammatory cells; (b) representative image of myocardial apoptosis detected using TUNEL staining (scale bar, 20 *μ*m) and quantification of TUNEL-positive cell nuclei (*n* = 6); (c) representative immunoblotting analysis and quantification of MKRN1, Bcl-2, Bax, Bak, p21, cleaved caspase-3, caspase-3 protein levels, and Bax/Bcl-2 ratio in the heart (*n* = 6) and GAPDH as an internal control; (d) IHC staining for Bcl-2, Bax, Bak, caspase-3, cleaved caspase-3, and p21 proteins in the heart (scale bar, 100 *μ*m); (e) MKRN1 mRNA expression in the heart was detected using qPCR (*n* = 6); (f) IHC staining for MKRN1 expression in the heart (scale bar, 100 *μ*m). *n* indicates the independent sample number in each group. ^∗∗^*p* < 0.01.

We conducted an in vivo experiment to demonstrate that IH significantly induced myocardial apoptosis, as well as performed HE staining to observe the effect of IH on rat myocardium. The cardiomyocytes in NC rats were structurally ordered, whereas those in IH rats were disorganized, with inflammatory cell infiltration (Figure 2(a)). We performed a TUNEL assay to analyze the impact of IH on rat myocardial apoptosis, which showed a significantly higher rate in IH rats compared to NC rats (Figure 2(b)). Then, we detected the expression of apoptosis-related proteins in myocardial tissues of NC and IH rats by WB and IHC. Compared to NC rats, the expression levels of the proapoptotic proteins Bax, Bak, cleaved caspase-3, p21, and Bax/Bcl-2 ratio significantly increased in the myocardial tissues of IH rats, whereas that of antiapoptotic Bcl-2 decreased (Figures 2(c) and 2(d)).

In the rat IH model, we further detected the changes in MKRN1 mRNA and protein expression in myocardial tissues using qPCR, IHC, and WB analyses. The MKRN1 mRNA and protein expression levels were significantly lower in rats in the IH group than in rats in the NC group (Figures 2(e) and 2(f)).

**Figure 3:**
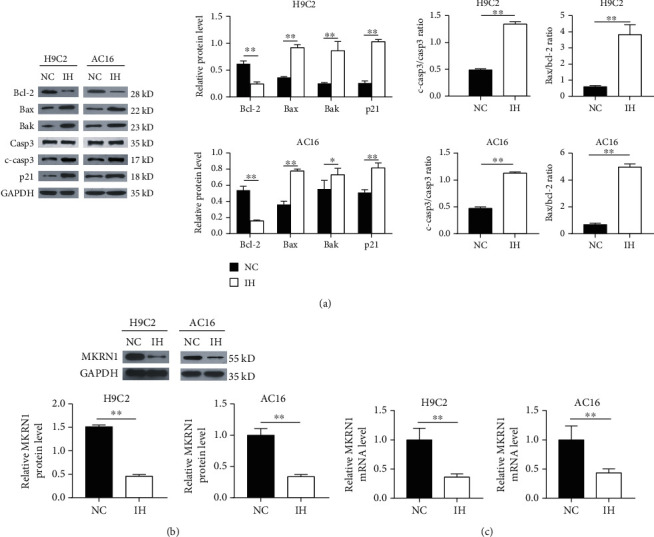
IH induces myocardial apoptosis and downregulates MKRN1 H9C2 and AC16 cells were subjected to NC or IH stimulation. (a) Representative immunoblotting analysis and quantification of Bcl-2, Bax, Bak, p21, cleaved caspase-3, caspase-3 protein levels, and Bax/Bcl-2 ratio (*n* = 3) and GAPDH as an internal control; (b) representative immunoblotting analysis and quantification of MKRN1 protein expression (*n* = 3) and GAPDH as an internal control; (c) MKRN1 mRNA expression was detected using qPCR (*n* = 3). *n* indicates the independent sample number in each group. ^∗∗^*p* < 0.01.

Then, we verified the abovementioned research results using in vitro experiments. We initially constructed IH cell models using rat cardiomyocytes (H9C2 cells) and human cardiomyocytes (AC16 cells). Consistent with results obtained from the rat IH myocardial tissues, IH upregulated Bax, Bak, cleaved caspase-3, p21 protein expression, and Bax/Bcl-2 ratio in H9C2 and AC16 cells and downregulated the Bcl-2 protein expression level (Figure 3(a)). These results indicated that IH promoted myocardial apoptosis. We further investigated the effect of IH on MKRN1 expression using the cell model. Compared to NC cells, MKRN1 mRNA and protein expression were reduced in H9C2 and AC16 cells in the IH group (Figures 3(b) and 3(c)).

### 3.3. MKRN1 Overexpression Alleviated IH-Induced ROS Production and Myocardial Apoptosis

**Figure 4:**
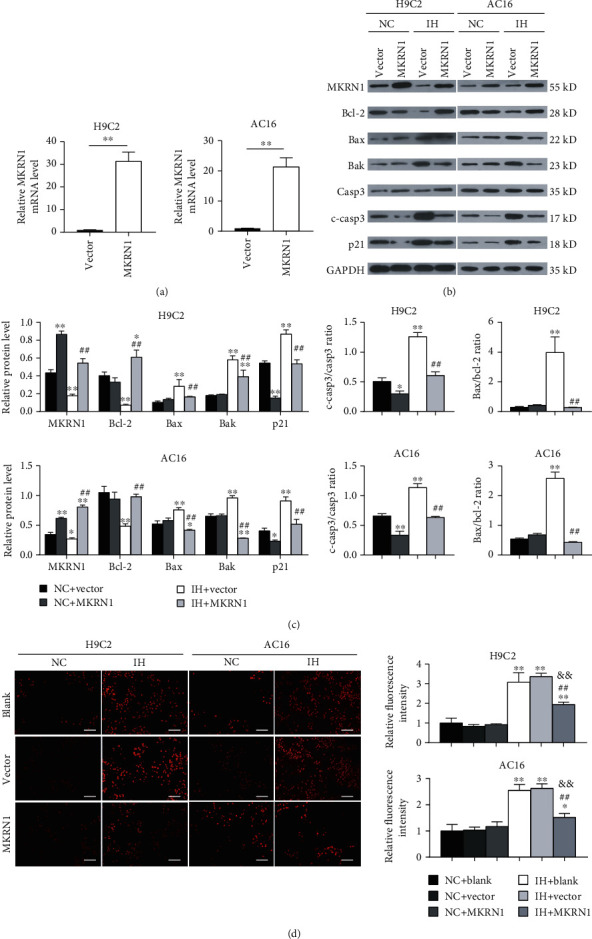
MKRN1 overexpression reduced IH-induced ROS production and myocardial apoptosis. H9C2 and AC16 cells transfected with MKRN1-overexpressing lentiviruses or empty virus were subjected to NC or IH stimulation. (a) MKRN1 mRNA expression was detected using qPCR (*n* = 3); (b, c) representative immunoblotting analysis (b) and quantification of MKRN1, Bcl-2, Bax, Bak, p21, cleaved caspase-3, caspase-3 protein levels, and the Bax/Bcl-2 ratio (c) (*n* = 3) and GAPDH as an internal control, ^∗^*p* < 0.05 and ^∗∗^*p* < 0.01 versus the NC + vector group; ^##^*p* < 0.01 versus the IH + vector group; (d) representative fluorescence image of DHE staining and quantification of fluorescence intensity (scale bar, 100 *μ*m) (*n* = 3), ^∗^*p* < 0.05 and ^∗∗^*p* < 0.01 versus the NC + blank group; ^##^*p* < 0.01 versus the IH + blank group; ^&&^*p* < 0.01 versus the IH + vector group. *n* indicates the independent sample number in each group.

To illustrate the effect of MKRN1 on IH-induced ROS production and myocardial apoptosis in vitro, we transfected H9C2 and AC16 cells with MKRN1-overexpressing lentiviruses or empty viruses to construct MKRN1-overexpressing cardiomyocytes. MKRN1-overexpressing lentiviruses significantly increased MKRN1 mRNA and protein levels (Figures 4(a)–4(c)). In NC cells, MKRN1 overexpression remarkably reduced the expression of the apoptosis marker, cleaved caspase-3, but it had little influence on the expression of Bcl-2 family members and the bax/bcl-2 ratio, and it significantly decreased the expression of the apoptosis regulatory protein, p21. In IH-subjected cells, MKRN1 overexpression evidently decreased the expression levels of p21 and cleaved caspase-3 and simultaneously increased the expression of Bcl-2 and decreased those of Bax, Bak, and the Bax/Bcl-2 ratio (Figures 4(b) and 4(c)). Excessive ROS production is considered an important cause of cell apoptosis, with superoxide ion (O_2_^•−^) acting as the main contributor. Therefore, we measured intracellular ROS production using DHE to detect O_2_^•−^. Compared to the cells in the NC group, IH treatment significantly induced stronger intracellular fluorescence intensities in H9C2 and AC16 cells, indicating higher ROS levels in cardiomyocytes subjected to IH. MKRN1 overexpression did not affect ROS production in the NC group, but it significantly decreased the ROS levels after IH stimulation (Figure 4(d)).

### 3.4. MKRN1 Expression Alleviated IH-Induced Myocardial Apoptosis in a p21-Dependent Manner

**Figure 5:**
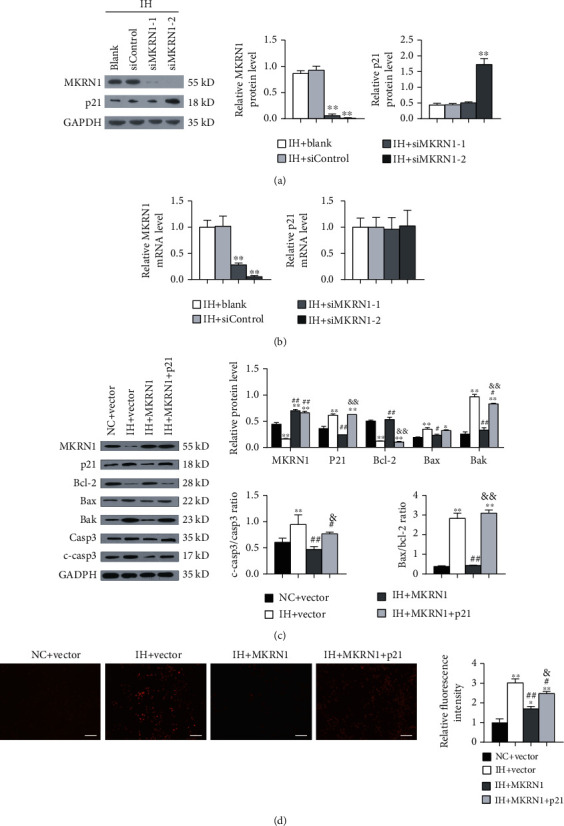
The effect of MKRN1 overexpression in cardiomyocytes is reversed by p21 overexpression under IH conditions. (a, b) H9C2 cells were transfected with control siRNA (siControl) or two kinds of MKRN1-siRNAs (siMKRN1-1 and siMKRN1-2) before IH stimulation. Thereafter, the (a) protein and (b) mRNA expression levels of MKRN1 and p21 were detected using WB and qPCR, respectively (*n* = 3); ^∗∗^*p* < 0.01 versus the IH + blank group; (c, d) H9C2 cells were transfected with the vector, MKRN1-overexpressing lentiviruses, and MKRN1-overexpressing lentiviruses combined with p21-overexpressing lentiviruses before IH stimulation. (c) Representative immunoblotting analysis and quantification of MKRN1, p21, Bcl-2, Bax, Bak, caspase-3, cleaved caspase-3 protein levels, and the Bax/Bcl-2 ratio (*n* = 3) and GAPDH as an internal control; ^∗^*p* < 0.05, ^∗∗^*p* < 0.01 versus the NC + vector group, ^#^*p* < 0.05, ^##^*p* < 0.01 versus the IH + vector group, ^&^*p* < 0.05, and ^&&^*p* < 0.01 versus the IH + MKRN1 group; (d) representative fluorescence image of DHE staining and quantification of fluorescence intensity (scale bar, 100 *μ*m) (*n* = 3); ^∗^*p* < 0.05, ^∗∗^*p* < 0.01 versus the NC + vector group, ^#^*p* < 0.05, ^##^*p* < 0.01 versus the IH + vector group, and ^&^*p* < 0.05 versus the IH + MKRN1 group. *n* indicates the independent sample number in each group.

To further investigate the effect of MKRN1 and the involvement of p21 in IH injury, we transfected MKRN1-siRNA into H9C2 cells for IH stimulation. Therefore, transfection with siMKRN1-1 and siMKRN1-2 remarkably suppressed the MKRN1 mRNA and protein expression and H9C2 cells transfected with siMKRN1-2 had the lowest MKRN1 expression. Hence, siMKRN1-2 was used in our subsequent experiments. Contrary to results from the overexpression experiments, MKRN1 protein was almost undetectable after MKRN1 silencing and p21 protein levels were markedly elevated (Figure 5(a)). Noteworthily, MKRN1 silencing resulted in a decreased MKRN1 mRNA expression level but it did not significantly change the p21 mRNA level (Figure 5(b)). This result suggested that, under IH condition, MKRN1 might negatively affect p21 protein stability. To verify whether the protection of MKRN1 against the IH-induced myocardial injury was related to p21, we conducted a rescue experiment. H9C2 cells were transfected with MKRN1-overexpressing lentiviruses, empty virus, and MKRN1-overexpressing lentiviruses combined with p21-overexpressing lentiviruses, before IH stimulation. Under IH conditions, p21 overexpression almost entirely reversed the effect of MKRN1 overexpression on the expression of Bcl-2, Bax, and the Bax/Bcl-2 ratio; in addition, it weakened the impacts on Bak, cleaved caspase-3 expression, and ROS production (Figures 5(c) and 5(d)). These results suggested that MKRN1 downregulated p21 protein expression to protect against IH-induced ROS production and myocardial apoptosis.

### 3.5. Under IH Conditions, MKRN1 Induced p21 Ubiquitination and Proteasome Pathway Degradation

**Figure 6:**
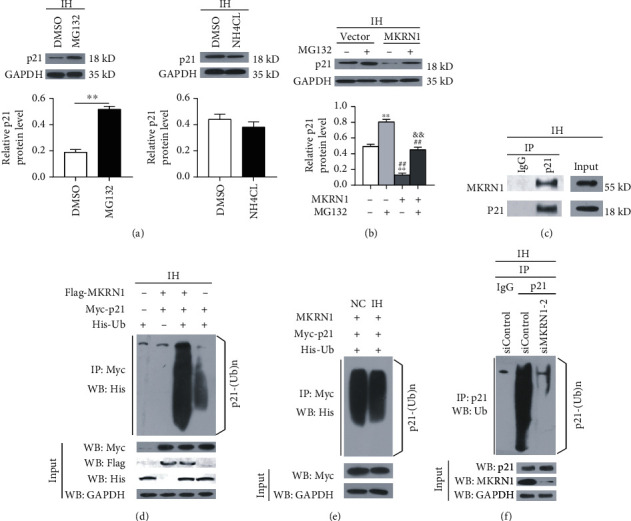
Under IH conditions, MKRN1 induced p21 ubiquitination and proteasome pathway degradation. (a) H9C2 cells were subjected to IH stimulation. Before harvest, cells were treated with DMSO, 20 *μ*M MG132 ((a), 1), or 25 mmol/L NH_4_Cl ((a), 2) for 3 h and representative immunoblotting analysis and quantification of p21 (*n* = 3), GAPDH as an internal control; ^∗∗^*p* < 0.01; (b) H9C2 cells were transfected with MKRN1-overexpressing lentiviruses or vector; before harvest, cells were treated with or without 20 *μ*M MG132 for 3 h and representative immunoblotting analysis and quantification of p21 (*n* = 3) and GAPDH as an internal control; ^∗∗^*p* < 0.01 versus the blank group, ^##^*p* < 0.01 versus the MG132 group, ^&&^*p* < 0.01 versus the MKRN1 group; (c) H9C2 cells were subjected to IH stimulation. Cell lysate and normal rabbit IgG antibody or p21 antibody were subjected to CO-IP, and the indicated antibodies were used for WB analysis; (d) 293T cells were transfected with plasmids expressing Flag-MKRN1, Myc-p21, and His-Ub before IH stimulation. Before harvest, cells were treated with MG132 for 3 h, the Myc antibody was used to purify Myc-p21 using CO-IP, and the His antibody was used for WB analysis; (e) cells in Figure 6(d) were subjected to NC or IH stimulation; before harvest, cells were treated with MG132 for 3 h, the Myc antibody was used for CO-IP, and the His antibody was used for WB analysis; (f) H9C2 cells transfected with siControl or siMKRN1-2 were subjected to IH stimulation, and MG132 was added 6 h prior to harvest. The whole-cell lysate was subjected to CO-IP using the p21 antibody, and the Ub antibody was used for WB analysis (the second and third columns), with normal rabbit IgG as the negative control (the first column). *n* indicates the independent sample number in each group.

To investigate the mechanism of MKRN1 in negatively regulating p21, we evaluated the p21 protein degradation pathway. Under IH conditions, the proteasome inhibitor MG132 suppressed p21 protein degradation, whereas the lysosome inhibitor NH_4_CL did not (Figure 6(a)), suggesting that p21 protein was degraded by the proteasome pathway and not the lysosome pathway, under IH status. In addition, MG132 treatment reversed the reduced p21 protein expression level induced by MKRN1 overexpression (Figure 6(b)), indicating that MKRN1 regulated p21 protein expression in a proteasome-dependent manner under IH conditions. Subsequently, we investigated whether the p21 ubiquitination process was regulated by MKRN1 under IH conditions. Based on CO-IP analysis, we observed that under IH conditions, the endogenous MKRN1 in H9C2 cells interacted with the endogenous p21 (Figure 6(c)). Then, we cotransfected 293T cells with plasmids expressing Flag-MKRN1, Myc-p21, and His-Ub before IH stimulation to detect the p21 protein ubiquitination level. The results showed that exogenous MKRN1 induced p21 protein ubiquitination under IH conditions (Figure 6(d)) and there was no significant difference in the function of MKRN1 in inducing p21 protein ubiquitination between NC and IH conditions (Figure 6(e)). Finally, we studied the effect of MKRN1 on endogenous p21 protein ubiquitination in H9C2 cells under IH conditions. As a result, the endogenous p21 protein ubiquitination level in MKRN1-depleted H9C2 cells was reduced (Figure 6(f)), revealing that MKRN1 mediated the endogenous p21 protein ubiquitination process in IH cardiomyocytes. Overall, our data suggested that, in the case of IH, MKRN1 promoted p21 protein ubiquitination, induced its proteasome pathway degradation, and, thus, negatively regulated p21 protein expression in cardiomyocytes.

## 4. Discussion

Myocardial apoptosis is the early manifestation of IH-induced myocardial injury [2], ROS plays an important role in cardiovascular injury, and inhibition of ROS can effectively alleviate cardiovascular injury. ROS-dependent neutrophil extracellular trap formation can induce inflammation of arterial endothelial cell, which is essential for promoting the progression of arteritis induced by selenium deficiency. Coculture of selenoprotein silencing arterial endothelial cells and neutrophils increased neutrophil extracellular trap formation, while acetylcysteine reversed the increase of neutrophil extracellular trap formation induced by selenoprotein silencing by inhibiting ROS burst [15]. Similarly, excessive ROS production plays a critical role in IH-induced myocardial apoptosis; suppressing ROS production can protect against IH-induced myocardial injury [16, 17]. Consistent with the results of previous studies [16], our results suggested that IH induced disorders in the myocardial structure and inflammatory cell infiltration and significantly increased myocardial apoptosis. Cell experiments further verified that IH stimulation increased ROS production in cardiomyocytes. Therefore, it is necessary to explore the mechanism of IH-induced ROS production and apoptosis promotion to develop improved prevention and treatment approaches for OSA-related myocardial injury.

IH stimulation can induce changes in the expression of multiple genes [18]. Through bioinformatics analysis, we discovered the abnormal MKRN1 expression, which was closely related to the apoptosis pathway. In addition, we further verified that IH induced the downregulation of MKRN1 in myocardial tissues and cells.

It has been shown that MKRN1 plays a crucial role in preventing excessive cell apoptosis by promoting FADD substrate ubiquitination and proteasome pathway degradation to delay the activation of the cell death receptor apoptosis pathway, reduce the sensitivity of cells to death ligands, and ultimately protect against cell death. Even in the absence of death ligand stimulation, the death receptor pathway apoptosis of MKRN1-depleted cells will also be activated [11]. However, the role of MKRN1 in myocardial apoptosis remains unclear at present. Our data suggested that MKRN1 overexpression suppressed IH-induced ROS production, alleviated the imbalanced expression of Bcl-2 family proteins, and significantly protected against IH-induced myocardial apoptosis. Interestingly, MKRN1 overexpression also suppressed myocardial apoptosis under normoxia conditions, without affecting ROS production. This suggests that MKRN1 can reduce ROS production and suppress IH-induced myocardial apoptosis, as well as inhibit myocardial apoptosis under normoxia via other mechanisms.

Apoptosis regulatory factor, p21, was assessed to determine the mechanism by which MKRN1 regulated ROS production and myocardial apoptosis. p21 is the downstream target molecule of MKRN1, which is negatively regulated by MKRN1. Under stress conditions induced by Adriamycin or ultraviolet stimulation, p21 can mediate the apoptosis regulatory effect of MKRN1 [19]. Concurrently, p21 is the upstream regulatory factor for hypoxia-induced myocardial apoptosis and ROS production. p21 overexpression can promote hypoxia-induced caspase-3 lysis and myocardial apoptosis [20]. Moreover, reducing miR-208 expression can upregulate the target gene p21 to promote ROS production, upregulate Bax expression, downregulate Bcl-2 expression, and aggravate ischemia-reperfusion-induced myocardial apoptosis [21]. In the case of severe cell injury, there is a positive feedback loop between p21 and ROS production and the injury can induce the persistent activation of the checkpoint gene p21, thus inducing mitochondrial dysfunction and ROS production, which, in turn, further activates p21 [22]. Consequently, we speculated that MKRN1 might protect against IH-induced myocardial injury through p21. We discovered that in IH myocardial tissues, p21 overexpression reversed the effect of MKRN1 overexpression on ROS production and apoptosis-related protein expression, suggesting that MKRN1 downregulated p21 expression to protect against IH-induced myocardial injury.

P21 is an unstable protein, and the ubiquitination-mediated proteasome pathway degradation is of crucial importance to maintain p21 stability [23, 24]. We discovered that MKRN1 negatively regulated p21 protein expression without affecting p21 mRNA levels, suggesting that MKRN1 might reduce p21 protein stability. Moreover, under IH conditions, p21 protein was still degraded through the proteasome pathway. MKRN1 is a kind of E3 ubiquitin ligase that exerts its biological effects through the ubiquitination modification of substrates. The role of MKRN1 in mediating p21 ubiquitination has been verified under physiological conditions and DNA-damage conditions; however, it remains unclear as to whether MKRN1 binding to substrates and its effect on promoting their ubiquitination are affected by the external environment [19]. Our results verified that, under IH conditions, MKRN1 interacted with p21, promoted p21 ubiquitination, induced its proteasome pathway degradation, and, thus, downregulated p21 protein expression in myocardial tissues. Moreover, under IH conditions, the level of MKRN1-induced p21 ubiquitination was the same as that under NC conditions. Collectively, our results indicated that, under IH conditions, MKRN1 negatively regulated p21 expression in cardiomyocytes through the ubiquitination of p21 protein and the promotion of its proteasome pathway degradation.

This experiment is aimed at evaluating the role and molecular action of MKRN1 in ROS production and myocardial apoptosis. Nonetheless, certain limitations should be noted in this study. First, the mechanism by which IH induced myocardial MKRN1 downregulation remains unclear. Second, this experiment only showed the effect of MKRN1 on resisting myocardial apoptosis through cell experiments. More animal and human studies are needed to further evaluate the potential of MKRN1 as a therapeutic target for the treatment of OSA-related myocardial injury.

Overall, this study establishes that MKRN1 expression is downregulated in myocardial tissues under IH condition. Additionally, MKRN1 promotes p21 ubiquitination and proteasome pathway degradation to downregulate p21 expression, thus reducing IH-induced ROS production and myocardial apoptosis. This study provides a new target for reducing cardiovascular risk among OSA patients.

## Data Availability

The data used to support the findings of this study are included within the article.
